# Automated Digital Notification of COVID-19 Diagnoses Through Text and Email Messaging — North Carolina, December 2020–January 2021

**DOI:** 10.15585/mmwr.mm7046a3

**Published:** 2021-11-19

**Authors:** Laura Farrell, Crystal R. Almond, Deborah S. Porterfield, Victoria Mobley, Sydney A. Jones, Marina Smelyanskaya, Erika Samoff

**Affiliations:** ^1^North Carolina Department of Health and Human Services; ^2^Resolve to Save Lives, New York, New York.

During October 3, 2020–January 9, 2021, North Carolina experienced a 400% increase in daily reported COVID-19 cases (*1*). To handle the increased number of cases and rapidly notify persons receiving a positive SARS-CoV-2 test result (patients), North Carolina state and local health departments moved from telephone call notification only to telephone call plus automated text and email notification (digital notification) beginning on December 24, 2020. Overall, among 200,258 patients, 142,975 (71%) were notified by telephone call or digital notification within the actionable period (10 days from their diagnosis date)* during January 2021, including at least 112,543 (56%) notified within 24 hours of report to North Carolina state and local health departments, a significantly higher proportion than the 25,905 of 175,979 (15%) notified within 24 hours during the preceding month (p<0.001). Differences in text notification by age, race, and ethnicity were observed. Automated digital notification is a feasible, rapid and efficient method to support timely outreach to patients, provide guidance on how to isolate, access resources, inform close contacts, and increase the efficiency of case investigation staff members.

Positive SARS-CoV-2 testing results are reported to North Carolina state and local health departments and managed in the North Carolina COVID-19 Surveillance System (NCCOVID)^†^ software. Before December 24, 2020, patients were notified through telephone calls by North Carolina case investigation staff members. On December 24, 2020, NCCOVID began electronically transferring case information (including patient name, positive laboratory test result, contact information, and date of birth) to the COVID-19 Community Team Outreach (CCTO)^§^ software used for contact tracing. Each case reported to NCCOVID within 10 days of the diagnosis date and with a documented telephone number or email address was automatically imported to the CCTO software. The CCTO software then triggered a text or email message alerting the patient of an important message about their COVID-19 test result with a website link and state call center telephone number. The website, which was only accessible via the notification link, provided the same information as in a telephone call: information about the positive test result, guidance on isolation, instructions on informing close contacts, and telephone numbers to call for assistance, including the state call center.

Patient text message statuses were grouped into four categories: 1) delivered (texts recorded as “sent” or “delivered”); 2) delivery status not recorded (texts with no final delivery status returned before the record was closed in the CCTO software); 3) undelivered (texts recorded as “failed” or “undelivered”); and 4) no valid phone number (texts not attempted because of missing or invalid phone number). To understand the likely final text status for texts in which the final delivery status was not recorded, aggregate data provided by text message service provider Twilio Inc. on final delivery status for all texts sent by the CCTO software, including those for purposes outside of digital case notification, were evaluated. These aggregate data could not be linked to individual CCTO software records. Delivery information for emails was not recorded in the CCTO software; emails were presumed to have been delivered. Patients were considered to have been digitally notified if a text was categorized as delivered or an email was sent.

Descriptive and inferential statistics were used to evaluate the impact of automated digital notification on notification timeliness (patients notified within 24 hours of report to North Carolina state and local health departments and notification completeness (patients notified within an actionable time frame; i.e., 10 days from diagnosis) in January 2021. The percentage of patients reached by digital notification or telephone call within 24 hours of report to North Carolina state and local health departments and within 10 days of diagnosis were compared before (November 23–December 23, 2020) and after (January 1–31, 2021) full implementation of automated digital notification. Information on timeliness and completeness for telephone notification was collected from staff member data entry in NCCOVID, and for digital notification, from system-generated timestamps in the CCTO software.^¶^ Records for which the time between specimen collection and notification dates was negative were treated as errors and removed from analyses.** A chi-square test was used to assess differences overall and by age, race, and ethnicity among patients not reached by text message. P-values <0.05 were considered statistically significant. Results were generated using SAS software (version 9.4; SAS Institute, Inc.). The project was determined to be a public health program evaluation and all applicable policies were followed.^††^

In January 2021, a positive SARS-CoV-2 test result was reported in NCCOVID for 200,258 patients (Figure 1). Among these, 172,274 (86%) records with a valid telephone number (including 39,928 that also had an email address) were transferred into the CCTO software, triggering a digital notification by text or email. Among all patients reported in NCCOVID, including those without a valid telephone number, a delivered text was recorded for 121,875 (61%) patients, a text delivery status was not recorded for 34,024 (17%) patients, and an undelivered text was recorded for 16,375 (8%) patients. Emails were sent to 40,923 (20%) patients. Among these, 3,468 (8% of emails and 1.7% of patients) were sent to patients who did not have documentation of a delivered text message, including 183 with a delivery status not recorded, 2,290 with a text outcome of undelivered and 995 with no valid telephone phone number. A separate analysis of aggregate Twilio data for all texts sent from the CCTO software in January 2021, including those for purposes outside of digital case notification, showed 89% delivered and 11% undelivered.

Overall, 125,343 patients (63%) during January 2021 were digitally notified (121,875 by text and 3,468 by email alone). During this time frame, the state call center received 14,616 incoming calls from patients, an increase of approximately 200% from the 4,933 calls received during the previous month. During January 24–31 (the only week during this period for which website data are available), 26,060 patients were digitally notified; this resulted in 54,747 visits to the notification website.

Among the January 2021 records, information on race and ethnicity was missing for 22% and 42% of patients, respectively. Among records with available race and ethnicity data, the percentage of patients not reached by text notification differed by race, ethnicity, and age group (Figure 2). Overall, 20% of Black patients and 22% of White patients were not reached (p<0.001), a higher percentage of non-Hispanic than Hispanic patients were not reached (21% and 13%, respectively) (p<0.001), and a higher percentage of American Indian or Alaska Native patients were not reached compared with all other races combined (26% versus 22%, respectively; p<0.001). Among patients aged ≥65 years, 39% were not reached compared with 19% of patients aged <65 years (p<0.001).

After implementation of digital notification, 112,543 of 200,258 (56%) patients were notified (by telephone call or digital notification) within 24 hours of report to North Carolina state and local health departments during January 2021, compared with 25,905 of 175,979 (15%) during the preceding month (p<0.001). Overall, 142,975 (71%) patients were notified within 10 days of their diagnosis date the month after implementation compared with 65,243 of 175,066 (37%) during the preceding month.

## Discussion

Patient notification of diagnosis and counsel to isolate is a critical component of COVID-19 control efforts; however, its impact on reducing COVID-19 transmission is diminished if diagnosis notification and patient isolation are delayed (*2*). Because of a surge in cases and an acute shortage of case investigation staff members, notifying patients by telephone was delayed. Implementation of automated digital notification enabled more timely notification of SARS-CoV-2 testing results, leading to approximately one half of patients being notified within 24 hours of report of the positive test result to North Carolina state and local health departments, compared with approximately one in six reached within 24 hours before implementation. Data indicated approximately twice as many clicks to the notification website (accessible only via the notification link) as the number of patients notified, suggesting a high level of engagement with the message. Research into engagement with this kind of landing page would generate useful information for improvement. These findings suggest that automated digital notification is a feasible, rapid, and efficient method that can be used to reach patients with COVID-19 in a timely manner.

Differences in text notification by age, race, and ethnicity were observed, suggesting that automated notification might not reach all groups equally. In this analysis, fewer older patients were successfully reached; this digital communication disparity among older adults has been reported previously (*3*). Since older adults and American Indian and Alaska Native persons experience less successful digital notification and more severe COVID-19 outcomes (*4*), telephone or field-based communication should be prioritized for these populations, and future studies might further evaluate how they can be better reached. Programs using this technology should ensure that the notification text delivery status is easily viewable by the case investigation staff members and should prioritize telephone or field-based communication for all patients for whom a notification text is undelivered. 

Exposure notification applications that identify contacts by time and proximity have been highlighted to mitigate COVID-19 by decreasing time to isolation among contacts that become infected and allowing rapid anonymous notification of contacts (*5*,*6*). However, use of these applications has been limited (*7*). Digital notification from surveillance systems can also decrease time to patient isolation via rapid notification of diagnosis results. Although this process cannot notify unknown contacts who have been in proximity to the patient, it avoids privacy concerns generated by location-sensing applications. In addition, although automated digital notification does not necessarily result in an increased proportion of patients isolating, it can decrease time to isolation, as supported by modeling studies (*5*,*8*), and provide information on accessing treatment. Therefore, there might be opportunities to improve disease control by expanding automated communication from surveillance systems. Future studies investigating whether automated digital notification leads to reduced secondary transmission because of earlier isolation are warranted.

The findings in this report are subject to at least three limitations. First, delivery status was unavailable for emails; therefore, the proportion of patients reached by email might be overstated because emails were assumed to have been delivered. Conversely, those reached by text might be understated because patients with an unrecorded text delivery status were not considered digitally notified; aggregate data from Twilio suggested that 89% of all texts were delivered. Second, data on race and ethnicity were missing for 22% and 42% of patients, respectively; complete data might identify different notification patterns. Finally, because patient isolation was not evaluated, the impact of automated digital notification on secondary infection remains unknown.

Automated digital notification of COVID-19 diagnosis is feasible and public health organizations that incorporate automated digital notification into their surveillance systems might reach patients with COVID-19 in a more timely fashion than can be achieved by telephone notification. In addition, enabling patients to provide close contact information digitally might also facilitate rapid notification of known contacts.^§§^ This automated notification has the potential to support rapid control of variant or other case surges; the technology is applicable to many diseases and would be beneficial for public health programs moving forward.

**Summary**

**What is already known about this topic?**

North Carolina implemented an automated digital notification system on December 24, 2020, to reach persons with diagnosed COVID-19 in a timely manner.

**What is added by this report?**

Overall, 56% of patients with a positive SARS-CoV-2 test result were notified by telephone call or digital notification within 24 hours of report in January 2021, compared with 15% during November 23–December 23, 2020. Differences in text notification by age, race, and ethnicity were observed.

**What are the implications for public health practice?**

Automated digital notification can provide a more timely means for reaching persons with COVID-19 and can likely facilitate more rapid patient isolation and increase efficiency of case investigation.

**FIGURE 1:**
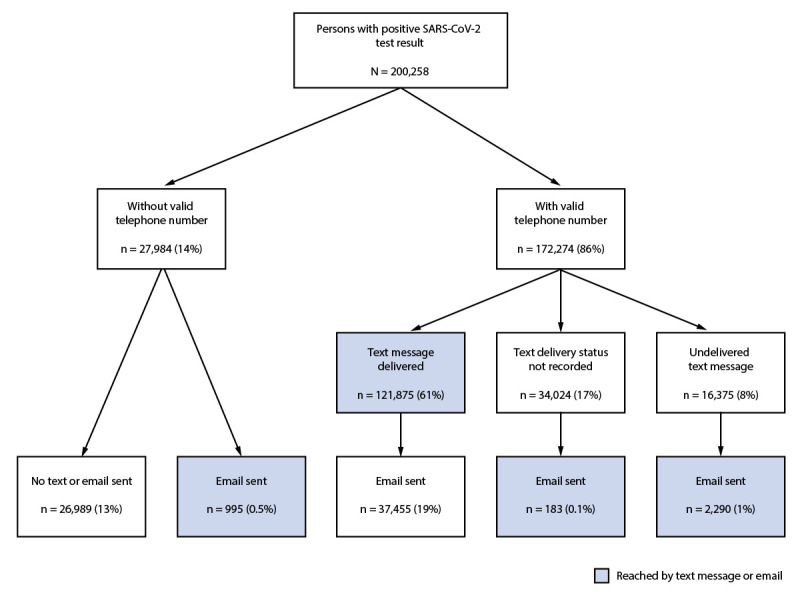
Notification status* of text messages and emails sent to persons with diagnosed COVID-19^†^ — North Carolina, January 2021 **Abbreviation:** CCTO = COVID-19 Community Team Outreach. * Based on data recorded in the CCTO contact tracing software. Delivered = texts recorded as “sent” or “delivered”; delivery status not recorded = texts with no final delivery status returned before the record was closed in the CCTO software; undelivered = texts that were recorded as “failed” or “undelivered”; no valid telephone number = no valid telephone number in surveillance records; email sent = email was sent (delivery confirmation unavailable in the CCTO software). ^†^ Positive SARS-CoV-2 reverse transcription–polymerase chain reaction or antigen test result reported to North Carolina Department of Health and Human Services.

**FIGURE 2:**
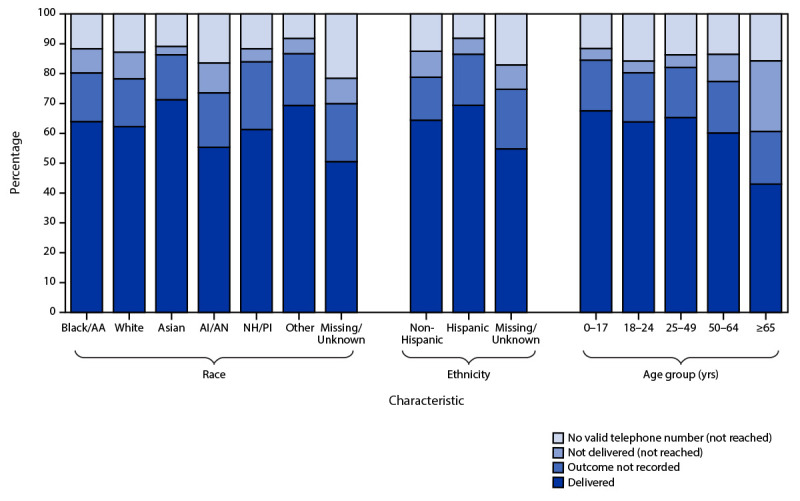
Notification status* of text messages sent to persons with diagnosed COVID-19 (N = 200,258),^†^ by race, ethnicity, and age group^§^ — North Carolina, January 2021 **Abbreviations:** AA = African American; AI/AN = American Indian or Alaska Native; CCTO = COVID-19 Community Team Outreach; NH/PI = Native Hawaiian or Other Pacific Islander. * Based on data recorded in the CCTO software. Delivered = texts recorded as “sent” or “delivered”; delivery status not recorded = texts with no final delivery status returned before the record was closed in the CCTO software; undelivered = texts recorded as “failed” or “undelivered”; no valid telephone number = no valid telephone number in surveillance records. ^†^ Positive SARS-CoV-2 reverse transcription–polymerase chain reaction or antigen test result reported to North Carolina Department of Health and Human Services. ^§^ As recorded in the North Carolina COVID-19 Surveillance System.

## Footnotes

* Diagnosis date refers to the date of collection of the first specimen with a positive SARS-CoV-2 test result for each COVID-19 event. Ten days was selected because it represents the period during which the patient is most likely to be infectious.

§ CCTO software is a customized Microsoft Dynamics software (Microsoft) that has the capacity to send automated texts and emails to persons from within its database.

** n = 933 during November 23–December 23, 2020; n = 244 during January 2021.

†† North Carolina Department of Public Health institutional review board review is not required for public health evaluation projects with no research component.
